# Surgical Triage of Breast Disorders During the COVID-19 Pandemic: An Indian Perspective

**DOI:** 10.21315/mjms2020.27.6.16

**Published:** 2020-12-29

**Authors:** Raghav Yelamanchi, Himanshu Agrawal, C K Durga

**Affiliations:** Department of Surgery, Post Graduate Institute of Medical Education and Research and Dr. Ram Manohar Lohia Hospital, New Delhi, India

**Keywords:** breast disorder triage, COVID-19 pandemic, breast surgery, resource management, India and developing countries

## Abstract

Breast complaints are a very common cause of healthcare visits in the female population. They range in severity from benign to malignant, and treatment options vary from simple observation to mastectomy. As healthcare facilities are overburdened with coronavirus disease 2019 (COVID-19) patients, properly triaging patients diagnosed with breast disorders is necessary for the optimal use of limited resources in developing countries. We are proposing a concise triage system for timely intervention among patients with breast disorders during the havoc of the COVID-19 pandemic.

Dear Editor-in-Chief,

Breast disorders are common among all age groups of females. Patients with breast disorders are often apprehensive due to fear of malignancy, which results in them seeking medical attention. All patients with breast complaints should be evaluated thoroughly and diagnosed in a healthcare setting. Currently, India is experiencing rapid growth in the number of coronavirus disease 2019 (COVID-19) cases post-lockdown. Consequently, intensive care units and wards are filled with COVID-19 patients, leading to a scarcity of resources for managing non-COVID-19 patients. Additionally, patients undergoing surgical procedures during this pandemic are at an increased risk of contracting the virus from the healthcare facilities (1). Therefore, decisions must be taken considering the risk-benefit ratio.

Triaging patients with breast disorders is necessary to provide the best possible care to patients who deserve the most, keeping in mind the limited manpower and resources. The most important factor to be considered while triaging patients is the impact of surgery on the outcomes and prognoses of patients. Governing bodies worldwide have come up with guidelines on triaging patients during this pandemic (2, 3). As India is different from its counterparts with respect to patient load, availability of healthcare facilities and socioeconomic background of patients, it needs to adopt these guidelines with modifications to suit its population. Hence, we recommend triaging patients into the following three categories.

## Category A

Patients who need emergent surgical intervention. This includes:

- Breast abscesses in septic patients
- Post-operative complications such as haematoma and wound infection
- Post-operative breast prosthesis and flap complications such as ischaemia and necrosis
- Breast cancer during pregnancy
- Breast cancer progressing during neoadjuvant therapy
- Patients with locally advanced breast cancer that is not suitable for any neoadjuvant therapy
- Locally advanced breast cancer with severe local complications such as ulceration, fungation and pain

## Category B

Patients whom surgical intervention cannot be delayed for more than 6 weeks. This includes:

- Patients who have finished neoadjuvant chemotherapy and are awaiting surgery
- Patients with early breast cancer that is not suitable for any neoadjuvant chemotherapy
- Local tumour recurrence in follow-up patients with breast cancer

## Category C

Patients whom surgical procedures can be delayed until the pandemic ends. This includes:

- Hormone-receptor-positive low-grade ductal carcinoma in situ
- Post-menopausal hormone-receptor-positive stage 1 and 2 breast cancers on endocrine therapy
- Patients who have undergone mastectomy and are awaiting delayed breast reconstruction
- Benign breast disorders such as fibroadenomas and duct disorders
- Mammoplasty procedures
- Prophylactic mastectomy

It should be noted that the above triage system should only be used during the peak of the pandemic. The emphasis is to use standard established guidelines whenever possible or as soon as the conditions return to normal.
